# Swept-source optical coherence tomography angiography of retinal occlusive vasculitis following brolucizumab administration: a case report

**DOI:** 10.1186/s12886-022-02465-0

**Published:** 2022-06-03

**Authors:** Eun Kyoung Lee, Baek-Lok Oh, Chang Ki Yoon, Un Chul Park

**Affiliations:** Department of Ophthalmology, Seoul National University College of Medicine, Seoul National University Hospital, #101, Daehak-ro, Jongno-gu, Seoul, Republic of Korea

**Keywords:** Age-related macular degeneration, Brolucizumab, Intraocular inflammation, Retinal occlusive vasculitis, Swept-source optical coherence tomography angiography

## Abstract

**Background:**

We present a case of retinal occlusive vasculitis following brolucizumab administration and the first report of optical coherence tomography angiography (OCTA) findings after treatment.

**Case presentation:**

A 71-year-old man complained of vision loss in the left eye 6 weeks after brolucizumab injection. His visual acuity was counting fingers, and examination revealed 1 + anterior chamber cells with 2 + vitreous cells. Fundus examination demonstrated vitreous haze, retinal whitening, and vascular sheathing. Fluorescein angiography revealed filling defects in the retinal arteries and veins, and OCTA showed extensive capillary nonperfusion. Under the diagnosis of brolucizumab-associated intraocular inflammation (IOI) and retinal occlusive vasculitis, topical, sub-Tenon, and systemic corticosteroids were administered. After the treatment, visual acuity improved to 20/200, and OCTA revealed gradual improvement in capillary dropout; however, with the limited improvement of reperfusion in the perifoveal areas.

**Conclusions:**

Prompt evaluation and intensive corticosteroid treatments are required for brolucizumab-associated IOI. OCTA imaging provides detailed information on microvascular changes in the retinal vascular plexuses in brolucizumab-associated retinal occlusive vasculitis.

## Background

Brolucizumab, a humanized, single-chain variable fragment that inhibits vascular endothelial growth factor (VEGF)-A was approved by the US Food and Drug Administration for treatment of neovascular age-related macular degeneration (AMD) on October 7, 2019 [1]. The phase 3 data from two pivotal clinical trials, HAWK (NCT02307682) and HARRIER (NCT02434328), demonstrated non-inferiority in best-corrected visual acuity (BCVA) with brolucizumab (6 mg/0.05 mL dosed at every 8 or 12 weeks) compared to aflibercept (2 mg/0.05 mL dosed at every 8 weeks) [2, 3]. Increased molar concentration combined with a high binding affinity for VEGF have been postulated to account for its potential for increased durability [4].

In terms of safety, HAWK and HARRIER studies found that the incidence of intraocular inflammation (IOI) was higher with brolucizumab having 4% compared to aflibercept’s 1%, and most of these cases were reported as mild to moderate [5, 6]. However, since the approval of brolucizumab, there were reports of severe visual acuity loss associated with retinal vasculitis and/or retinal artery occlusion accompanied by IOI following treatment with brolucizumab [7–10]. Novartis announced that the incidence rate of retinal vasculitis and/or retinal vascular occlusion was estimated to be 15.5 per 10,000 injections on post-marketing reports [11]. To further clarify the incidence of these events, Novartis commissioned an external Safety Review Committee (SRC) [11, 12]. The SRC conducted a post hoc unmasked analysis of the images from cases in HAWK and HARRIER that were reported as having IOI. They concluded that the incidence of IOI was 4.6% (IOI + retinal vasculitis, 3.3%; IOI + retinal vasculitis + retinal vascular occlusion, 2.1%), and the overall incidence of at least moderate vision loss associated with IOI was 0.74% [12].

Little is known about the mechanism of IOI and patient characterization and approaches to reduce these adverse events have not yet been clearly established. Given that the number of reported case series of retinal occlusive vasculitis is highly limited, a better understanding of this condition requires more information. Furthermore, to our knowledge, optical coherence tomography angiography (OCTA) in patients with retinal occlusive vasculitis associated with brolucizumab injection has not yet been reported. Herein, we report a case of retinal occlusive vasculitis after intravitreal brolucizumab administration and present the OCTA findings after treatment.

### Case presentation

A 71-year-old man with active neovascular AMD in the left eye returned to the clinic owing to a significantly decreased vision in the left eye (OS). The patient was systemically healthy and did not report a history of diabetes or hypertension. Surgical history was significant only for cataract extraction with a posterior chamber intraocular lens in OS. He had been treated with six intravitreal ranibizumab injections and 25 intravitreal aflibercept injections since 2014 for typical AMD in OS. There was persistent subretinal fluid (SRF) despite multiple injections of ranibizumab and aflibercept. He had never experienced any IOI with either medication. The decision to switch to brolucizumab was made given the persistent SRF in the OS.

**Fig. 1:**
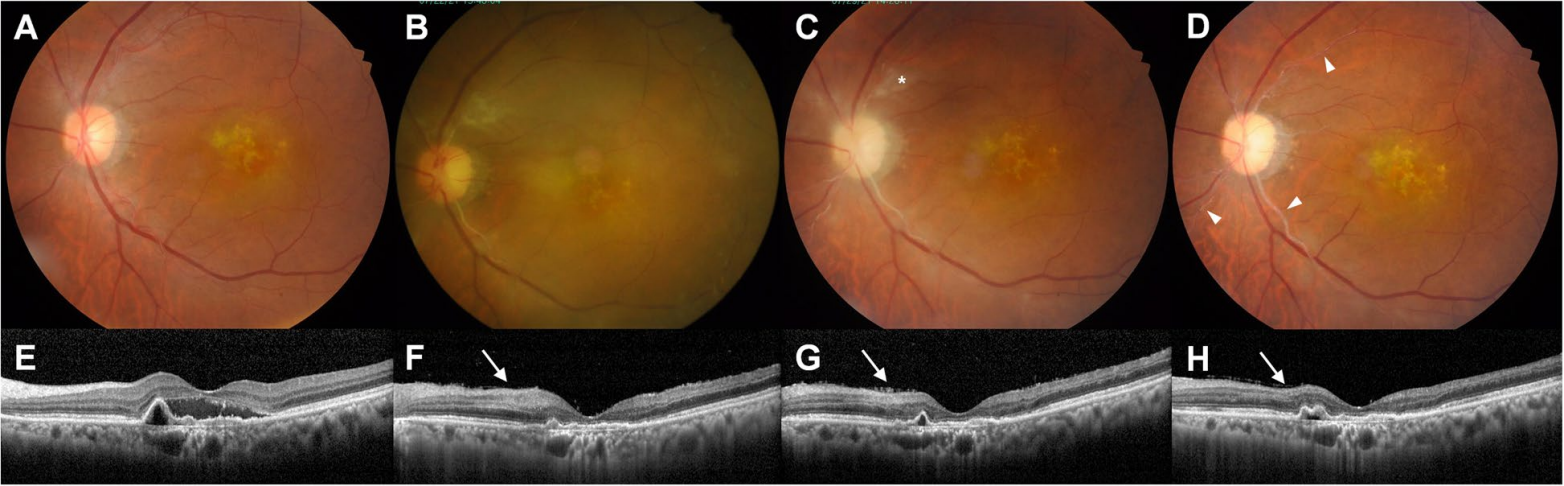
Fundus photographs (**A** **-** **D**) and cross-sectional horizontal optical coherence tomography (OCT) B-scan images (**E** **-** **H**). All images are arranged from the leftmost column in the order of before (**A**, **E**), 6 weeks (**B**, **F**)**-**development of intraocular inflammation (IOI)-, 7 weeks (**C**, **G**), and 12 weeks (**D**, **H**) after the brolucizumab administration. After the development of IOI and retinal occlusive vasculitis, cotton-wool patches (white asterisk) suggesting precapillary retinal arteriolar occlusion and retinal whitening gradually decreased over time; however, Kyrieleis plaques (white arrowheads) remained. On OCT images, a hyperreflective band consistent with paracentral acute middle maculopathy (white arrows) suggesting inner nuclear layer ischemia decreased and pigment epithelial detachment increased over time

**Fig. 2:**
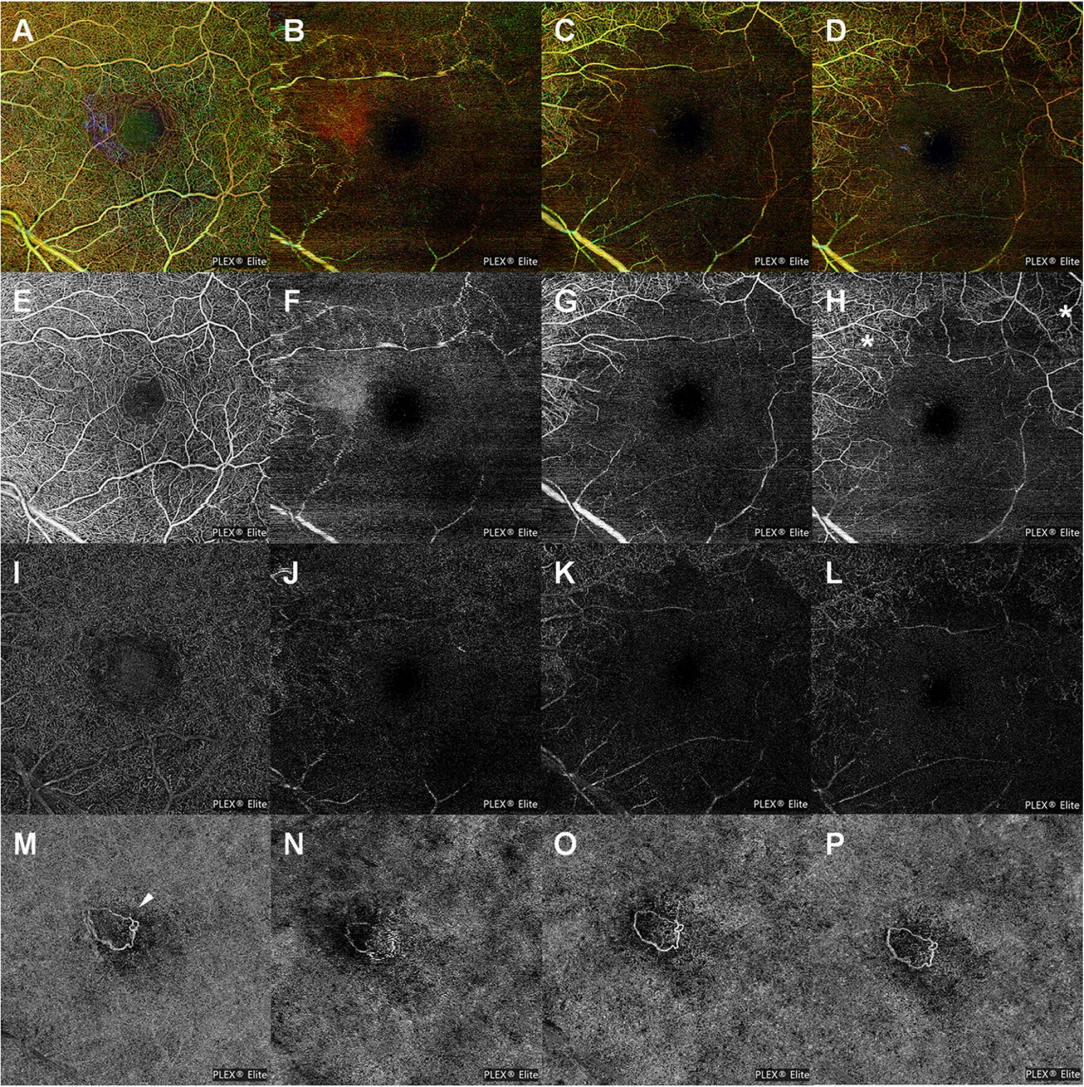
Swept-source optical coherence tomography angiography (OCTA) images that correspond to the retina depth-encoded (**A** **-** **D**), superficial capillary plexus (SCP) (**E** **-** **H**), deep capillary plexus (DCP) (**I** **-** **L**), and outer retina to choriocapillaris (ORCC) slab (**M** **-** **P**). All images are arranged from the leftmost column in the order of before (**A**, **E**, **I**, **M**), 6 weeks (**B**, **F**, **J**, **N**)-development of intraocular inflammation (IOI)-, 7 weeks (**C**, **G**, **K**, **O**), and 12 weeks (**D**, **H**, **L**, **P**) after the brolucizumab administration. On OCTA images, extensive non-perfusion areas with capillary dropout were apparent in SCP and DCP. Following the treatment, recanalized capillaries (white asterisks) are shown; however, with persistent perifoveal nonperfusion. The shape and size of choroidal neovascularization (white arrowhead) reveal no specific changes in ORCC slabs

**Fig. 3:**
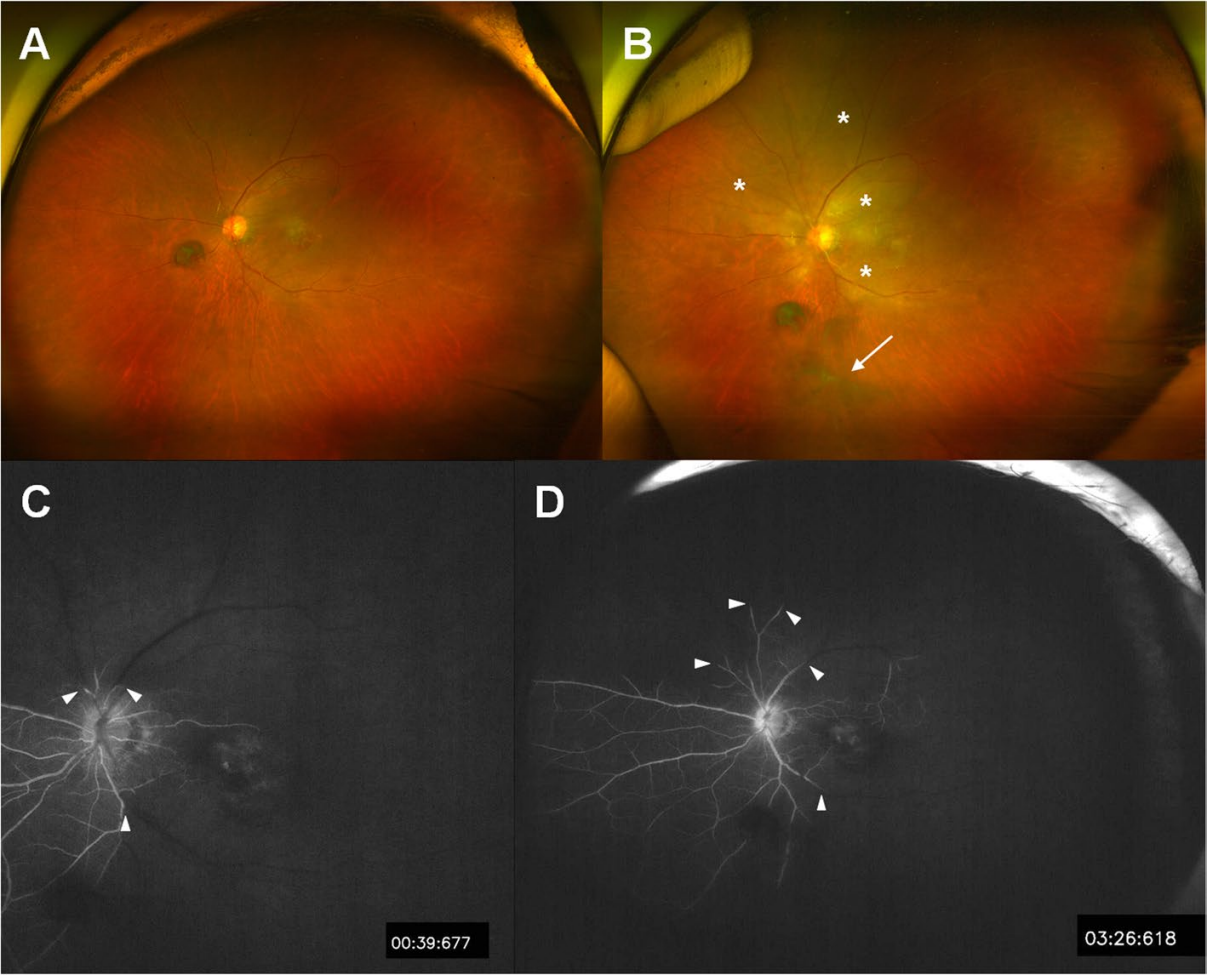
Ultra-widefield fundus photography (**A**, **B**) and ultra-widefield fluorescein angiography (**C**, **D**) recorded 2 weeks (**A**) and 6 weeks (**B** **-** **D**) after the brolucizumab administration. **A** No intraocular inflammation is reported. **B** Vitreous opacity (white arrow), retinal whitening (white asterisks) suggestive of associated ischemia in this area are shown. **C**, **D** Fluorescein angiography demonstrating arterial and venous filling defects (white arrowheads), which persisted into the late phase and involved the macula

On examination before brolucizumab injection, his BCVA was 20/20 in the right eye (OD) and 20/100 in the OS. Fundus examination revealed choroidal neovascularization (CNV) with serous detachment in the OS (Fig. 1A). Optical coherence tomography (OCT) showed foveal SRF with pigment epithelial detachment (PED) (Fig. 1E). The swept-source OCTA device (PLEX Elite 9000; Carl Zeiss Meditec, Inc., Dublin, CA) was used to evaluate the neovascular networks in terms of their location, shape, size, and extent. OCTA revealed no specific vascular network changes at the level of both the superficial and deep capillary plexus and detects CNV by the presence of an abnormal pattern of vascular flow in the outer retina to the choriocapillaris (ORCC) slab (Fig. 2A, E, I, and M). Two weeks after the first brolucizumab injection, BCVA was 20/125 and intraocular pressure (IOP) was within normal limits in OS. Slit-lamp examination was normal, and ultra-widefield fundus photography revealed nonspecific findings (Fig. 3A).

Six weeks after the brolucizumab injection, the patient experienced visual deterioration, which started 3 days back. On clinical examination, his BCVA was counting fingers, and the IOP was 13 mmHg in OS. Slit-lamp examination showed anterior chamber cells (1 +) with fine keratic precipitates and vitreous cells (2 +); however, no hypopyon. Fundus examination showed vitreous haze, retinal whitening, cotton-wool patches, and vascular sheathing involving the major retinal arteries, all of which were suggestive of IOI (Fig. 1B). On OCT imaging, SRF and PED were decreased; however, inner retinal edema was observed (Fig. 1F). OCTA showed extensive non-perfusion areas in the superficial capillary plexus, as well as diffuse capillary network attenuation and disorganization in the deep capillary plexus (Fig. 2B, F, J, N). Ultra-widefield fundus photography revealed vitreous opacity, vascular sheathing, and retinal whitening, particularly in the superotemporal, superonasal, and inferotemporal quadrants (Fig. 3B). Ultra-widefield fluorescein angiography (FA) showed filling defects in the larger retinal arteries and veins proximal to the optic nerve, which remained until late-phase FA, as well as optic disc staining (Fig. 3C, D).

Based on the clinical features and history of brolucizumab, a diagnosis of brolucizumab-associated IOI and retinal occlusive vasculitis was made. The patient was treated with oral prednisolone 30 mg daily and topical prednisolone acetate 1.0% (Pred-forte®) every 2 h. Four days later, he was administered an additional sub-Tenon triamcinolone acetonide injection. One week after the diagnosis of retinal occlusive vasculitis, his BCVA was 20/400 and IOP was 14 mmHg in OS. Slit-lamp examination showed anterior chamber cells (trace) and vitreous cells (1 +); however, no keratic precipitates. Fundus examination showed decreased vitreous haze (Fig. 1C), and OCT revealed decreased inner retinal edema (Fig. 1G). OCTA demonstrated a slight improvement in areas of capillary nonperfusion in both superficial and deep capillary plexuses and no obvious changes in CNV in the ORCC slab (Fig. 2C, G, K, O). Aspirin 100 mg/day and clopidogrel 75 mg/day were additionally prescribed for the patient.

Three weeks after the diagnosis of retinal occlusive vasculitis, his BCVA was 20/200, and the IOP was 14 mmHg. Slit-lamp examination revealed anterior chamber cells (trace) and vitreous cells (trace). Fundus examination revealed that vitreous haze, cotton-wool patches, and retinal whitening decreased more; however, vascular sheathing involving the retinal arteries remained and optic disc pallor was noted (Fig. 1D). On OCT imaging, inner retinal edema decreased further, and there was no recurrence of SRF; however, PED gradually increased (Fig. 1H). OCTA showed gradual improvement in capillary dropout in both superficial and deep capillary plexuses and no obvious changes in CNV in the ORCC slab (Fig. 2D, H, L, P). However, the perifoveal areas revealed no apparent reperfusion and limited improvement throughout the follow-up period. The dose of oral prednisolone was tapered gradually by 2.5–5 mg every 1–2 weeks. Thereafter, the patient received an additional intravitreal aflibercept injection.

## Discussion and conclusion

This is the first report of brolucizumab-associated retinal occlusive vasculitis in Korean patients. In Korea, brolucizumab was approved by the Korean Ministry of Food and Drug Safety on June 15, 2020, and has been commercially available since April 1, 2021. Although the period of experiencing brolucizumab in Korea is still short, clinical data are accumulating, and there are still concerns regarding brolucizumab-associated IOI. We present a case of neovascular AMD in a 71-year-old man who developed IOI and retinal occlusive vasculitis after intravitreal brolucizumab using OCTA images. To the best of our knowledge, this is the first study to demonstrate the findings of OCTA in brolucizumab-associated retinal occlusive vasculitis.

Brolucizumab (~ 26 kDa) is the smallest of the anti-VEGF antibodies, significantly smaller than bevacizumab (149 kDa), aflibercept (97 − 115 kDa), and ranibizumab (48 kDa) [1]. Such a size difference gives brolucizumab theoretically better target-tissue penetration and therefore a higher concentration that allows up to 6 mg of brolucizumab in a single 50-µL intravitreal injection, resulting in an anti-VEGF binding capacity 11 and 22 times greater than that of aflibercept and ranibizumab, respectively [1]. The mechanism of retinal occlusive vasculitis and IOI after brolucizumab injection is still not clear; however, several hypotheses have been proposed. Some authors postulated that owing to its more potent anti-VEGF effect, brolucizumab may have a sufficiently high anti-VEGF effect to cause retinal arteriolar constriction and occlusive vasculopathy compared with other anti-VEGF agents [9]. Additionally, relatively late onset of IOI suggests that delayed hypersensitivity against the drug itself or impurities in the product may have been involved with brolucizumab-associated IOI [7, 13]. The European Medicines Agency’s suggestion that serum antibodies to the drug did correlate with IOI in the clinical trials also support that local antibodies to the drug may be associated with IOI development [14]. Local antibodies may form immune complexes leading to vasculitis through a mechanism of delayed hypersensitivity [7, 8, 10, 15]. Other suggested causes include the use of previous anti-VEGF therapy, previous IOI events, human leukocyte antigens, and comorbidities [7–10].

MERLIN study investigated the safety and efficacy of brolucizumab at 4 weekly intervals and observed that IOI was higher with brolucizumab (9.3%) compared to aflibercept (4.5%) [16]. Furthermore, proportion of patients with visual loss was also significantly higher with brolucizumab having 4.8% compared to aflibercept’s 1.7%. These results suggest that frequent exposure leads to higher immunogenicity. Sharma et al. [17] hypothesized that free brolucizumab molecules would likely accumulate in the vitreous if the injection frequency was less than a month, and that a significant amount could escape into the systemic circulation, leading to the formation of more anti-drug antibodies, thereby enhancing immunogenicity.

The management of brolucizumab-associated IOI in the HAWK and HARRIER trials generally included topical corticosteroids, and systemic and/or intraocular corticosteroids use was infrequent. However, the management approach in the recent literatures recommends more intensive corticosteroid treatment administered by a variety of methods including topical, sub-Tenon, oral, intravitreal, or a combination thereof [7, 9, 10, 18]. Based on the expert panel's recommendations, the eight-step "A BRAVE SAVE" protocol has been suggested [19]. This protocol recommends that intensive treatment of patients with brolucizumab-associated IOI should be started with potent topical corticosteroids. In patients with more severe IOI, intravitreal steroid injections along with systemic corticosteroid therapy should be considered. There have been post-marketing case reports in which brolucizumab-associated adverse events present as a spectrum in which IOI was the initial presentation and retinal vasculitis and/or retinal vascular occlusion developed subsequently in a delayed manner [7, 10]. Therefore, adequate inflammation control with potent corticosteroids may reduce the risk of progression to a more serious spectrum of IOI-related adverse events. Additionally, this intensive treatment could also minimize the risk of secondary ischemic changes after retinal vascular occlusive events [20].

OCTA can provide high-resolution images of individual vascular plexuses in different retinal layers in a noninvasive manner. Previous studies have shown that OCTA plays an important role in the diagnosis and treatment of uveitis [21–24]. OCTA appears to be particularly useful for a detailed evaluation of retinal nonperfusion as well as improvement of retinal ischemia after treatment. The changes observed in the present study support the observations of previous studies in that OCTA is useful in evaluating retinal nonperfusion and confirms that it provides exceptionally detailed images of microvascular flow changes in the retinal vascular plexuses in patients with brolucizumab-associated IOI and retinal occlusive vasculitis.

Although the patient in the current study was treated using intensive corticosteroids, visual acuity demonstrated limited improvement and complete reperfusion was not achieved on OCTA images at the latest follow-up. Although the patient was sufficiently educated about symptoms associated with IOI in advance, he visited the hospital with retinal vascular occlusion on the third day after symptom onset, which eventually resulted in irreversible visual acuity deterioration. Approximately 75% of the brolucizumab-associated IOI events were first observed within 6 months after initiation of brolucizumab, although some occurred between 12 and 18 months after the injection [12]. Therefore, after the first injection of brolucizumab, clinicians must be aware of the maximum inflammation risk soon after treatment. In particular, even if there is no evidence of inflammation 2 weeks after the first injection of brolucizumab, as in this patient, it is still not reassuring, and the patient must be followed-up for a shorter period after that. It is important to educate patients to visit the hospital as soon as they experience symptoms such as floaters, light sensitivity, or decreased vision. Ultra-widefield imaging techniques are also required to identify retinal vasculitis or retinal vascular occlusion.

In conclusion, a high index of suspicion is required when a patient presents with visual symptoms following brolucizumab treatment. Urgent and continued evaluation is necessary for patients with brolucizumab-associated IOI as occlusive vasculitis may develop subsequently. OCTA imaging is useful for the detailed evaluation of retinal nonperfusion in patients with brolucizumab-associated retinal occlusive vasculitis. Prompt and intensive treatment with topical, intraocular, and/or systemic corticosteroids to control inflammation may prevent further visual deterioration and may allow some improvement of vision in these patients.

## Data Availability

All data generated or analyzed during this study are included in this published article.

## Abbreviations

VEGF: Vascular endothelial growth factor
AMD: Age-related macular degeneration
BCVA: Best-corrected visual acuity
IOI: Intraocular inflammation
SRC: Safety Review Committee
OCTA: Optical coherence tomography angiography
OS: Oculus sinister
SRF: Subretinal fluid
OD: Oculus dextrus
CNV: Choroidal neovascularization
OCT: Optical coherence tomography
PED: Pigment epithelial detachment
ORCC: Outer retina to the choriocapillaris
IOP: Intraocular pressure
FA: Fluorescein angiography
